# Supplementary material for: Chronic Alcohol Consumption is Inversely Associated with Insulin Resistance and Fatty Liver in Japanese Males
Source: Nutrients. 2020 Apr 9;12(4):1036. doi: 10.3390/nu12041036 (PMC7231020; doi:10.3390/nu12041036)
Supplement: Supplementary file 1 [file nutrients-12-01036-s001.pdf]

Table S1. Stanardized questionnaire

Questions	
1. How often do you have a drink containing alcohol for the last year?	1. Never 2. times/week
2. How much do you drink on a typical day when you are drinking?	Beer ml/day<br>Sake ml/day<br>Shochu ml/day<br>Whiskey ml/day<br>Wine ml/day
3. Do you often drink sweet beverage?	1. Yes 2. No
4. Do you often eat greasy meat?	1. Yes 2. No
5. Are you in a habit of doing exercise to sweat lightly for over 30 minutes a time, 2 times weekly, for over a year?	1. Yes 2. No
